# Sex differences in documented clinical features of memory clinic patients: a natural language processing study

**DOI:** 10.1016/j.cccb.2026.100544

**Published:** 2026-04-23

**Authors:** Sanne Kuipers, L. Malin Overmars, Bram van Es, Sander C. Tan, Jet M.J. Vonk, Majon Muller, Mark C.H. de Groot, Wouter W. van Solinge, Saskia Haitjema, Michiel L. Bots, Geert Jan Biessels, Lieza G. Exalto

**Affiliations:** a Department of Neurology and Neurosurgery, UMC Utrecht Brain Centre, University Medical Center Utrecht, Utrecht University, Utrecht, the Netherlands; b Central Diagnostic Laboratory, University Medical Center Utrecht, Utrecht University, Utrecht, the Netherlands; c Department for Research & Data Technology, University Medical Center Utrecht, Utrecht, Utrecht University, the Netherlands; d Memory and Aging Center, Department of Neurology, University of California San Francisco, San Francisco, CA, USA; e Department of Internal Medicine and Geriatrics, Amsterdam UMC, location VUmc, Amsterdam, the Netherlands; f Julius Center, for Health Sciences and Primary Care, University Medical Center Utrecht, Utrecht University, Utrecht, the Netherlands

**Keywords:** Sex differences, Cognitive impairment, Memory clinic, Routine care data, Natural language processing, Clinical features, Machine learning

## Abstract

**Introduction:**

Sex differences in signs and symptoms of cognitive impairment are well described in research cohorts, but whether they are reflected in routine clinical documentation is unknown.

**Methods:**

We applied natural language processing to clinical letters from 915 memory clinic patients in the Utrecht Patient-Oriented Database. Sex differences in documented clinical features were assessed with logistic regression, with and without age adjustment. Benjamini–Hochberg correction was applied for multiple comparisons.

**Results:**

We included 1036 clinical letters from 915 patients (47% women; mean age 66 ± 14 vs. 67 ± 13 years, p = 0.6). The most common clinical features were memory problems (56.5% overall) and forgetting intended actions (49.6%). After correction for multiple comparisons, no significant sex differences remained. At the nominal level, women more often had documentation of stress (OR 0.39), headache (OR 0.63), and calendar use for memory support (OR 0.66); men more often had documentation of getting angry easily (OR 1.72) and short-term memory problems (OR 1.70).

**Discussion:**

This exploratory study identified suggestive but non-significant sex differences in documented clinical features in memory clinic practice. Differences may reflect variation in occurrence, reporting, or documentation habits, and warrant replication in larger, diagnosis-stratified samples.

## Highlights

• Natural language processing was applied to 1036 routine memory clinic letters to examine sex differences in documented clinical features.
• We found showed substantial overlap in documented clinical features between women and men, with modest differences in a small subset of features.
• No sex differences remained statistically significant after correction for multiple comparisons.
• Documented clinical features reflect a combination of symptom occurrence, patient reporting, and clinician documentation practices.
• The study demonstrates the applicability of NLP for investigating documentation patterns in routine clinical data.

## 1 Background

There is a growing body of literature that documents sex differences in clinical phenotypes of memory clinic patients, predominantly in Alzheimer’s dementia (AD) [1,2]. Women show a more rapid cognitive decline following a diagnosis of mild cognitive impairment (MCI) or AD, and women and men with AD may display different cognitive and psychiatric complaints [2]. For example, verbal memory is more preserved in women at early stages of AD relative to that in men with the same diagnosis [3].

Previous studies on sex differences in AD symptomatology have predominantly focused on differences in neuropsychiatric symptoms according to questionnaires [2,4]. A meta-analysis of 62 studies, including 21,554 patients with AD, found that women with AD experienced greater severity of depression, anxiety, psychotic symptoms (particularly delusions), and aberrant motor behavior, whereas men exhibited more severe apathy [5].

Although questionnaires used in observational cohort studies are comprehensive, they may differ significantly from clinical practice settings. Analyzing textual electronic health records (EHRs) using natural language processing (NLP)-techniques, therefore, offers complementary insights reflective of real-world clinical observations.

Recent studies have already shown promising results using NLP-techniques to analyze neuropsychiatric symptoms in the dementia field. In older adults with cognitive impairment NLP-techniques have been used to estimate the prevalence of neuropsychiatric symptoms from EHRs [6,7]. and identify adults at higher risk for dementia based on neuropsychiatric symptoms [8]. A recent study across two memory clinic cohorts also used NLP-techniques to estimate neuropsychiatric symptoms and found that clinicians recorded more neuropsychiatric symptoms in EHRs than caregivers reported on questionnaires (the neuropsychiatric inventory) [9].

This study aims to explore sex differences in recorded clinical features in clinical notes of memory clinic patients using NLP-techniques, providing real-world insight in the sex-differences in such clinical features recorded during routine clinical practice.

## 2 Methods

### 2.1 Data extraction

Clinical notes were extracted from the Utrecht Patient-Oriented Database (UPOD) for patients who visited the memory outpatient clinic at the University Medical Center Utrecht (UMC Utrecht) between November 2011 and August 2023. UPOD is an infrastructure of relational databases containing electronic health records (EHRs) for all individuals treated at UMC Utrecht [10]. The governance and usage of UPOD comply with the Institutional Review Board (IRB) and privacy regulations of UMC Utrecht, ensuring that clinical data are pseudonymized and that individuals may opt out of research use.

Identification was based on appointment codes used by the memory clinics: NGEH, NGEH+, NGEHP, NGEHV, and NVCI. Only correspondence documents labeled as either “Poliklinische Brief” (i.e. Outpatient letter) or “Klinische Brief” (i.e. Clinical letter) and containing the keyword “Anamnese” were retained for further analysis. A patient could have multiple clinical notes over time.

Of note, this study analyzes differences in recorded clinical features based on biological sex assigned at birth, as recorded in electronic health records (EHRs) based on government-issued identification.

### 2.2 Natural language processing

**Fig. 1:**
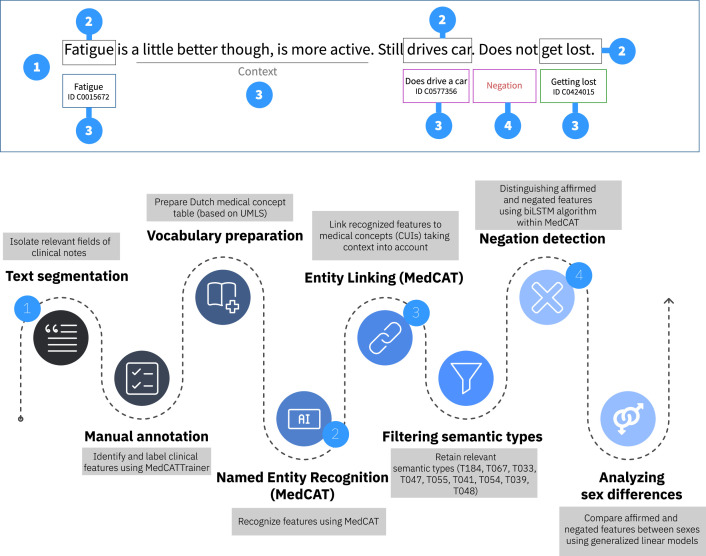
Stepwise overview of the clinical feature extraction process from clinical notes using MedCAT. Clinical notes were preprocessed and segmented. Entities were extracted via Named Entity Recognition and Linking, with contextual embeddings mapping entities to canonical concepts. Concepts were filtered by semantic type, and assertion status (affirmed/negated) was assigned using a biLSTM-based negation detector. Sex differences in affirmed and negated documented clinical features were estimated with logistic regression models (generalized linear models with binomial family and logit link).

The process of extracting documented clinical features from clinical notes involved a series of NLP-techniques, including preprocessing, segmentation, manual annotation, Named Entity Recognition and Linking (NER+L) using MedCAT [11], and semantic type filtering, summarized below (Fig. 1).

#### 2.2.1 Text segmentation

First, the texts were segmented to isolate the anamnesis sections from clinical notes. Specifically, the clinical text was split using the following regex patterns:

# Split the text into paragraphs using double newline as the delimiter paragraphs <- unlist(strsplit(vci$Brief_txt, "\\r\\n\\r\\n"))

# Keep only the paragraph that starts with "Anamnese" anamnese_paragraph <- paragraphs[grep("^Anamnese", paragraphs)]

#### 2.2.2 Manual annotation

Next, a subset of 200 clinical notes was manually annotated to explicitly identify and label documented clinical features, enriching the Dutch medical Concept Database (CDB) underlying MedCAT. This was done in MedCATTrainer [12].

#### 2.2.3 Vocabulary preparation

Next, we prepared a Dutch CDB that includes a list of medical concepts (clinical features, definitions, and unique identifiers) sourced from the Unified Medical Systems (UMLS; 2022AB release, comprising 254,835 concepts) [13]. The CDB was further augmented with phrases identified during manual annotation.

#### 2.2.4 Named Entity Recognition (NER) (MedCAT)

Named Entity Recognition (NER) is the process of identifying medically relevant terms (entities) within clinical text. MedCAT uses SpaCy embeddings to detect possible entities by analyzing the surrounding context. At this stage, MedCAT determines if a piece of text represents a medical entity but does not yet link it to a specific medical concept.

#### 2.2.5 Entity Linking (MedCAT)

Entity Linking is the subsequent process of associating recognized medical entities with specific concepts in the Concept Database (CDB). MedCAT resolves ambiguities by retrieving candidate concepts from the CDB and comparing their learned embeddings to the surrounding textual context using cosine similarity. For example, the entity "cold" could refer either to a viral infection or a low temperature. MedCAT uses contextual clues such as "runny nose" or "fever" to link the entity accurately to the appropriate medical concept.

#### 2.2.6 Filtering semantic types

Next, recognized and linked entities were then manually filtered to include only those corresponding to clinically relevant semantic types. The selected semantic types were: T184: Sign or Symptom, T067: Phenomenon or Process, T033: Finding, T047: Disease or Syndrome, T055: Individual Behavior, T041: Mental Process, T054: Social Behavior, T039: Physiologic Function, T048: Mental or Behavioral Dysfunction . Thus, the NLP pipeline extracts a heterogeneous set of items from clinical letters, including symptoms (e.g., headache), signs (e.g., getting lost), compensatory strategies (e.g., use of a calendar), and behavioral observations (e.g., oblivious). We use the term “clinical features” throughout to encompass this diversity.

#### 2.2.7 Negation detection

We distinguished between affirmed and negated documented clinical features using a biLSTM negation detection algorithm integrated within MedCAT [14]. For example, the symptom "getting lost" is affirmed in the phrase "she regularly gets lost," but negated in the phrase "she doesn’t get lost" or " no indications that Ms. gets lost".

### 2.3 Statistical analysis

Sex differences in documented clinical features were assessed for the top 25 most frequently occurring features, separately for affirmed and negated mentions. For each clinical feature, a binary variable was created at the level of the individual clinical letter, indicating whether the feature was documented (1) or not (0). Logistic regression models (generalized linear models with a binomial distribution and logit link function) were fitted with this binary indicator as the dependent variable and sex as the independent variable (women = reference). Results are reported as odds ratios (ORs) with 95 % confidence intervals (CIs) in forest plots. In addition to the unadjusted models, we fitted age-adjusted models including age at the time of the clinical letter as a covariate. To account for the multiple comparisons across 25 features, p-values were corrected using the Benjamini–Hochberg procedure for controlling the false discovery rate (FDR). Both uncorrected and FDR-corrected p-values are reported. Descriptive statistics (counts, percentages, percentage-point differences, and ratios) are presented for context.

The analyses were performed with Python version 3.10.7 and R version 4.4.2.

## 3 Results

A total of 1036 clinical letters from 915 patients were included. Women (n = 430, 47 %) had a mean age of 66 ± 14 years; men (n = 485, 53 %) had a mean age of 67 ± 13 years (p = 0.6).

**Fig. 2:**
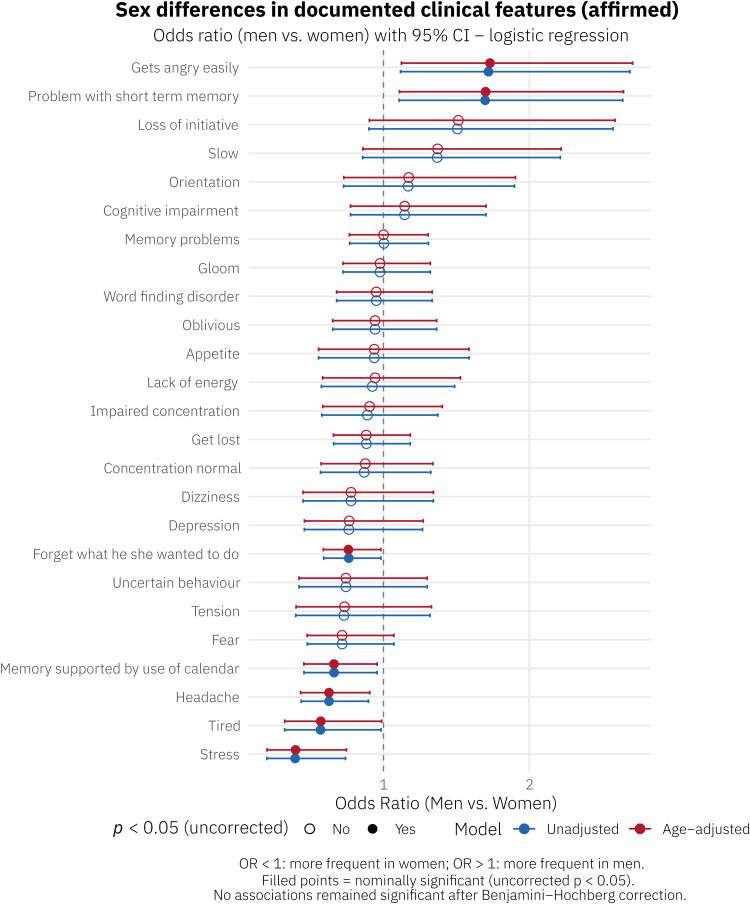
Sex differences in top 25 affirmed clinical features Sex differences in top 25 affirmed documented clinical features. Forest plot showing odds ratios (ORs) with 95 % confidence intervals for the association between sex (men vs. women) and documentation of each clinical feature, estimated by logistic regression. Blue circles: unadjusted models; red circles: age-adjusted models. Filled symbols indicate nominal significance (uncorrected p < 0.05). No associations remained significant after Benjamini–Hochberg correction. OR < 1: more frequent in women; OR > 1: more frequent in men.

Sex differences in the top 25 affirmed documented clinical features are shown in Table 1, Fig. 2, and Supplemental Table S1. The most frequently documented features were memory problems (57.4 % in women vs. 55.8 % in men) and forget what he/she wanted to do (52.7 % vs. 46.9 %) (Table 1). After Benjamini–Hochberg correction for multiple comparisons, none of the observed sex differences remained statistically significant (Fig. 2, Supplementary Table S1). At the nominal significance level (uncorrected p < 0.05), several suggestive patterns emerged. Documentation of stress was more frequent in women than in men (6.2 % vs. 2.3 %; OR 0.39, 95 % CI 0.20–0.74; p = 0.005) (Table 1, Fig. 2, Supplemental Table S1), as was headache (11.7 % vs. 8.1 %; OR 0.63, 95 % CI 0.44–0.90; p = 0.011), calendar use for memory support (15.4 % vs. 10.4 %; OR 0.66, 95 % CI 0.46–0.96; p = 0.029), forgetfulness (52.7 % vs. 46.9 %; OR 0.76, 95 % CI 0.59–0.98; p = 0.036), and tiredness (6.8 % vs. 4.2 %; OR 0.57, 95 % CI 0.32–0.98; p = 0.046). Conversely, getting angry easily (11.7 % vs. 6.8 %; OR 1.72, 95 % CI 1.12–2.69; p = 0.015) and short-term memory problems (OR 1.70, 95 % CI 1.11–2.64; p = 0.017) were documented more often in men. Age-adjusted results were highly consistent with the unadjusted findings (Fig. 2).

**Table 1:** Sex differences (women-to-men) in top 25 affirmed clinical features.

**Clinical feature**	**Overall n**	**Overall %**	**Women n**	**Women %**	**Men n**	**Men %**	**W–M Δ ( %)**	**%W / %M**
Memory problems	559	56.5	269	57.4	290	55.8	1.6	1.0
Forget what he/she wanted to do	491	49.6	247	52.7	244	46.9	5.7	1.1
Get lost	178	18.0	86	18.3	92	17.7	0.6	1.0
Gloom	131	13.2	60	12.8	71	13.7	−0.9	0.9
Oblivious	129	13.0	63	13.4	66	12.7	0.7	1.1
Memory supported by use of calendar	126	12.7	72	15.4	54	10.4	5.0	1.5
Word finding disorder	117	11.8	59	12.6	58	11.2	1.4	1.1
Cognitive impairment	105	10.6	48	10.2	57	11.0	−0.7	0.9
Headache	97	9.8	55	11.7	42	8.1	3.7	1.5
Gets angry easily	93	9.4	32	6.8	61	11.7	−4.9	0.6
Concentration normal	93	9.4	46	9.8	47	9.0	0.8	1.1
Impaired concentration	87	8.8	44	9.4	43	8.3	1.1	1.1
Fear	83	8.4	47	10.0	36	6.9	3.1	1.4
Slow	71	7.2	27	5.8	44	8.5	−2.7	0.7
Lack of energy	67	6.8	33	7.0	34	6.5	0.5	1.1
Orientation	64	6.5	26	5.5	38	7.3	−1.8	0.8
Loss of initiative	60	6.1	22	4.7	38	7.3	−2.6	0.6
Appetite	58	5.9	28	6.0	30	5.8	0.2	1.0
Tired	54	5.5	32	6.8	22	4.2	2.6	1.6
Depression	54	5.5	29	6.2	25	4.8	1.4	1.3
Uncertain behaviour	51	5.2	27	5.8	24	4.6	1.1	1.2
Dizziness	46	4.7	26	5.5	20	3.8	1.7	1.4
Tension	43	4.3	23	4.9	20	3.8	1.1	1.3
Stress	41	4.1	29	6.2	12	2.3	3.9	2.7

W–M Δ: women-to-men difference in percentage points. %W/ %M: ratio of percentages.

**Fig. 3:**
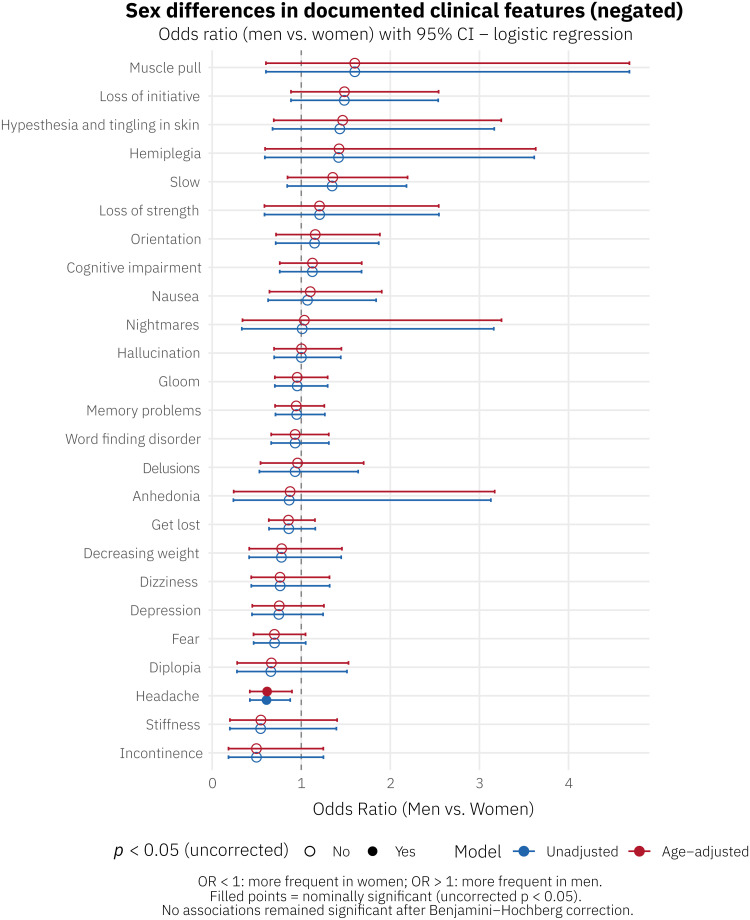
Sex differences in top 25 negated clinical features Sex differences in top 25 negated documented clinical features. Forest plot showing odds ratios (ORs) with 95 % confidence intervals for the association between sex (men vs. women) and documentation of each clinical feature, estimated by logistic regression. Blue circles: unadjusted models; red circles: age-adjusted models. Filled symbols indicate nominal significance (uncorrected p < 0.05). No associations remained significant after Benjamini–Hochberg correction. OR < 1: more frequent in women; OR > 1: more frequent in men.

Fig. 3 and Supplementary Table S2 display sex differences in the top 25 negated clinical features (i.e., features explicitly documented as absent). After correction for multiple comparisons, no significant sex differences were observed. At the nominal level, headache was the only negated feature with an uncorrected p < 0.05 (OR 0.61, 95 % CI 0.42–0.88; p = 0.008), indicating it was more frequently documented as absent in women's notes.

## 4 Discussion

Using NLP to extract clinical features from clinical letters, we identified sex differences in the documentation of clinical features among memory-clinic patients. Women more often had documentation of stress, headache, use of a calendar to support memory, and forgetfulness (“forget what she wanted to do”); notably, stress was documented about 2.4-fold more often in women. Men more often had documentation of getting angry easily and short-term memory problems.

It is important to note that the items extracted by the NLP pipeline represent a heterogeneous set of clinical features, including symptoms (e.g., headache), signs (e.g., getting lost), compensatory strategies (e.g., use of a calendar), and behavioral descriptors (e.g., oblivious). We use the encompassing term “clinical features” to reflect this diversity. The distinctions between seemingly similar items (e.g., “memory problems” versus “forget what he/she wanted to do”) arise from the granularity of the underlying medical terminology database (UMLS), to which MedCAT links each text fragment based on its surrounding context. Some overlap between these categories is likely, but merging them would sacrifice the nuance captured by the NLP pipeline.

In this study, we identified both overlap and differences in documented clinical features between women and men at a memory clinic. However, it remains speculative whether certain clinical features occur more frequently, are mentioned more often, or are more thoroughly inquired about or documented in one sex over the other. Various factors may influence the differences in documented clinical features. In general, previous literature shows that women report more symptoms than men, including both medically explained and unexplained complaints [15]. Also, collateral medical history (heteroanamnesis) provided by a proxy, often a partner of the opposite biological sex, may highlight different issues during the interview, as seen in reporting of neuropsychiatric symptoms [16]. Additionally, the healthcare provider conducting the assessment, and potentially even their sex/gender and origin, could play a role. Our finding that the symptom 'headache' was both more frequently affirmed and negated in women in unadjusted models is intriguing, suggesting it was inquired about or documented more often in women. Perhaps, the symptom of headache is more frequently inquired about in women at the memory clinic due to higher prevalence of headache among women. Nevertheless, it remains uncertain whether men experience this symptom less frequently or if it is simply inquired about less in men at the memory clinic.

Importantly, the overall pattern shows substantial overlap between women and men for the majority of documented clinical features. After correction for multiple comparisons, none of the observed sex differences remained statistically significant, which is consistent with the exploratory nature of this study and the modest effect sizes. The nominally significant associations — particularly the higher documentation of stress and headache in women, and of getting angry easily and short-term memory problems in men — may represent genuine sex-specific patterns, but require replication in larger, diagnosis-stratified samples before firm conclusions can be drawn.

Important limitations include that we analyzed all memory-clinic attendees irrespective of diagnosis; because symptom profiles are syndrome-specific and diagnostic distributions differ by sex (e.g., Alzheimer’s disease more common in women; behavioral-variant frontotemporal dementia more common in men), residual confounding is likely. Moreover, MedCAT may miss clinical features that are implied rather than explicitly stated (e.g., apathy described as loss of interest without the term “apathy”), which could lead to under-ascertainment; large language models might mitigate this contextual gap, but they are not currently permitted in our setting. A human concordance study would likely increase capture, although there is no clear reason to expect systematic sex-differential under-recognition. Taken together, these findings should be interpreted as differences in clinical documentation rather than definitive differences in symptom occurrence. The unit of analysis in this study is the individual clinical letter rather than the individual patient. Consequently, patients with multiple letters contribute multiple observations, which may influence the results. We chose this approach because aggregating to the patient level would require assumptions about how to summarize features that appear in some but not all of a patient’s letters, which we considered less transparent.

This study draws on routinely collected clinical data, which was generated for care purposes rather than for research. As a consequence, key clinical variables that would be relevant for stratification or adjustment are not uniformly available across patients. Diagnosis, for example, is often not yet established at the time of the anamnesis, as patients are typically referred to the memory clinic for diagnostic work-up; where a working diagnosis is recorded, it may appear as free text within letters rather than as a structured field. Education level is occasionally mentioned in the clinical narrative but is not a structured variable in the EHR. Comorbidities such as hypertension are typically managed by the general practitioner and may be documented in referral letters, but these were stored in a separate document management system and were not available within UPOD at the time of this analysis. Similarly, cognitive test scores are recorded in neuropsychological testing databases that were not linked to the current dataset. Furthermore, documentation practices have evolved over the study period (2011–2023), meaning that variables recorded in more recent letters may not be available for earlier patients. Including incompletely recorded covariates could introduce bias rather than reduce it. We were able to adjust for age, which is reliably and uniformly recorded, and found that age-adjusted results were largely consistent with the unadjusted findings. However, residual confounding due to unmeasured variables cannot be excluded. In particular, because we analyzed all memory clinic attendees irrespective of diagnosis, and clinical feature profiles are syndrome-specific while diagnostic distributions differ by sex, unmeasured diagnostic confounding may contribute to the observed patterns.

Despite, as discussed above, sex differences as recorded by NLP could be due to different causes or even biases, it is of interest to compare our findings to existing studies that used other techniques, particularly regarding neuropsychiatric symptoms. Most previous studies focused on neuropsychiatric symptoms in AD patients, often using questionnaire-based assessments [2], this hampers the comparison with our findings. These studies suggest that men with AD are more likely than women to exhibit clinical features such as apathy [17], agitation [18], and socially inappropriate or abusive behavior [17,19,20]. Conversely, women with AD tend to present more often with depressive symptoms [21], reclusiveness [17], emotional lability [17], delusions [22], and affective or manic symptoms [23]. A recent study using NLP-techniques to identify symptoms from the 12 domains of the Neuropsychiatric Inventory in EHRs of memory clinic patients with MCI, AD or mixed dementia, also examined sex differences in neuropsychiatric symptoms [9]. The study found that the clinical features agitation, aberrant motor behavior, apathy, disinhibition, irritability, and sleeping behavior were more often recorded in men, while the symptoms anxiety and depression were more recorded in women. Overall, our finding that men were more frequently identified with the symptom 'gets angry easily' compared to women aligns with existing literature. Our study extends prior research by examining sex differences across a broader range of general clinical features, beyond neuropsychiatric ones, using NLP-techniques in memory clinic patients.

The implications of this study are twofold. First, it highlights the potential of NLP for recognizing complaint patterns linked to specific patient characteristics. Second, it raises awareness of sex differences in clinical presentation of patients at memory clinics, helping healthcare providers recognize that women and men may report distinct clinical features. Certain complaints may also carry more significance for one sex than the other. By acknowledging these differences, care can be better tailored to individual patients. Moreover, they could serve as a basis for generating hypotheses on sex differences in clinical phenotypes, which are instrumental in the development of 'precision medicine' approaches in AD and memory complaints, encompassing sex-sensitive strategies for prevention, detection, drug development and treatment [2].

In conclusion, this exploratory study used NLP to examine sex differences in documented clinical features of memory clinic patients. While the overall pattern showed substantial overlap, suggestive differences emerged — with stress, headache, and forgetfulness documented more often in women, and getting angry easily and short-term memory problems more often in men — although none survived correction for multiple comparisons. These findings underscore the potential of NLP for recognizing documentation patterns linked to patient characteristics, and highlight the need for larger, diagnosis-stratified studies to establish whether sex-specific clinical feature profiles exist in memory clinic populations.

## AI statement

During the preparation of this work the author(s) used Claude (Anhtropic) in order to textually improve the manuscript. After using this tool/service, the author(s) reviewed and edited the content as needed and take(s) full responsibility for the content of the published article.

## Consent statement

The ethics of our study was reviewed by the Medical Ethics Committee NedMec (METC NedMec). METC NedMec is a recognized Medical Research Ethics Committee in the Netherlands, formed through a collaboration between UMC Utrecht, Prinses Máxima Centrum for pediatric oncology, and the Antoni van Leeuwenhoek institute. As our research involves retrospective analysis of electronic health records (EHR) and does not fall under the scope of the Dutch Medical Research Involving Human Subjects Act (WMO), the committee determined that formal ethical approval was not required (waived). Furthermore, in line with GDPR Article 14(5)(b), individual patient consent was not required due to the disproportionate effort involved in contacting all individuals. Data management specialists from an ISO 9001 certified database (Utrecht Patient-Oriented Database) extracted, pseudonymized and securely stored the data. As we worked with electronic health records collected with an IRB waiver for informed consent from NedMec, under the disproportionate effort clause, I will not be able to share data with others.

## Funding

This work is part of the Heart-Brain Connection crossroads (HBCx) consortium of the Dutch CardioVascular Alliance (DCVA). HBCx has received funding from the Dutch Heart Foundation under grant agreements 2018-28 and CVON 2012-06. Additionally, LMO was funded by funded by the European Union’s Horizon Europe Research and Innovation Program under Grant Agreement Nos. 101057849 (DataTools4Heart) and 101080430 (AI4HF), and TAP-Dementia, a ZonMW funded project (#10510032120003) part of the Dutch National Dementia Strategy.

## CRediT authorship contribution statement

**Sanne Kuipers:** Writing – original draft, Formal analysis, Conceptualization. **L. Malin Overmars:** Writing – original draft, Visualization, Software, Methodology, Investigation, Formal analysis, Conceptualization. **Bram van Es:** Writing – review & editing, Software, Methodology. **Sander C. Tan:** Writing – review & editing, Software, Methodology. **Jet M.J. Vonk:** Writing – review & editing. **Majon Muller:** Writing – review & editing. **Mark C.H. de Groot:** Writing – review & editing, Data curation. **Wouter W. van Solinge:** Writing – review & editing, Supervision. **Saskia Haitjema:** Writing – review & editing, Supervision, Funding acquisition. **Michiel L. Bots:** Writing – review & editing, Supervision. **Geert Jan Biessels:** Writing – review & editing, Supervision, Funding acquisition. **Lieza G. Exalto:** Writing – review & editing, Writing – original draft, Supervision, Funding acquisition, Conceptualization.

## Declaration of competing interest

The authors declare the following financial interests/personal relationships which may be considered as potential competing interests:

Malin Overmars reports financial support was provided by Dutch Heart Foundation. Lieza Exalto reports financial support was provided by Dutch Heart Foundation. Sanne Kuipers reports financial support was provided by Dutch Heart Foundation. Malin Overmars reports financial support was provided by European Union Horizon Europe Research and Innovation Program. Malin Overmars reports financial support was provided by NWO-ZonMW. If there are other authors, they declare that they have no known competing financial interests or personal relationships that could have appeared to influence the work reported in this paper.

## Appendix Supplementary materials

Image, application 1
