# Supplementary material for: Sex differences in documented clinical features of memory clinic patients: a natural language processing study
Source: Cereb Circ Cogn Behav. 2026 Apr 23;10:100544. doi: 10.1016/j.cccb.2026.100544 (PMC13142027; doi:10.1016/j.cccb.2026.100544)
Supplement: Supplementary file 1 [file mmc1.docx]

**Supplemental Material**

**Supplemental Table S1**

| Affirmed clinical features: Unadjusted logistic regression (sex M vs F) | | | | | | |
| --- | --- | --- | --- | --- | --- | --- |
| Odds ratios with Benjamini-Hochberg corrected p-values | | | | | | |
| **Clinical feature** | **OR** | **SE** | **p (unadj)** | **OR lower** | **OR upper** | **p (BH-adj)** |
| Stress | 0.39 | 0.33 | 0.01 | 0.20 | 0.74 | 0.10 |
| Headache | 0.63 | 0.18 | 0.01 | 0.43 | 0.89 | 0.10 |
| Gets angry easily | 1.72 | 0.22 | 0.01 | 1.11 | 2.69 | 0.10 |
| Problem with short term memory | 1.70 | 0.22 | 0.01 | 1.10 | 2.64 | 0.10 |
| Memory supported by use of calendar | 0.66 | 0.18 | 0.02 | 0.45 | 0.95 | 0.14 |
| Forget what he/she wanted to do | 0.76 | 0.13 | 0.03 | 0.59 | 0.98 | 0.15 |
| Tired | 0.56 | 0.28 | 0.04 | 0.32 | 0.98 | 0.16 |
| Fear | 0.71 | 0.20 | 0.10 | 0.47 | 1.07 | 0.32 |
| Loss of initiative | 1.50 | 0.26 | 0.12 | 0.90 | 2.57 | 0.34 |
| Slow | 1.36 | 0.24 | 0.19 | 0.85 | 2.21 | 0.48 |
| Tension | 0.72 | 0.30 | 0.29 | 0.39 | 1.31 | 0.57 |
| Depression | 0.76 | 0.25 | 0.29 | 0.45 | 1.26 | 0.57 |
| Uncertain behaviour | 0.74 | 0.28 | 0.29 | 0.42 | 1.30 | 0.57 |
| Dizziness | 0.77 | 0.27 | 0.36 | 0.44 | 1.34 | 0.65 |
| Get lost | 0.88 | 0.15 | 0.40 | 0.65 | 1.18 | 0.67 |
| Cognitive impairment | 1.14 | 0.20 | 0.50 | 0.77 | 1.70 | 0.72 |
| Concentration normal | 0.86 | 0.21 | 0.51 | 0.56 | 1.32 | 0.72 |
| Orientation | 1.16 | 0.24 | 0.52 | 0.72 | 1.89 | 0.72 |
| Impaired concentration | 0.89 | 0.22 | 0.59 | 0.57 | 1.37 | 0.78 |
| Lack of energy | 0.92 | 0.24 | 0.74 | 0.57 | 1.48 | 0.87 |
| Oblivious | 0.94 | 0.18 | 0.75 | 0.65 | 1.36 | 0.87 |
| Word finding disorder | 0.95 | 0.17 | 0.77 | 0.67 | 1.33 | 0.87 |
| Appetite | 0.93 | 0.26 | 0.80 | 0.55 | 1.58 | 0.87 |
| Gloom | 0.97 | 0.15 | 0.87 | 0.72 | 1.32 | 0.91 |
| Memory problems | 1.00 | 0.13 | 0.98 | 0.76 | 1.30 | 0.98 |

**Supplemental Table S2.**

| Negated clinical features: Unadjusted logistic regression (sex M vs F) | | | | | | |
| --- | --- | --- | --- | --- | --- | --- |
| Odds ratios with Benjamini-Hochberg corrected p-values | | | | | | |
| **Clinical feature** | **OR** | **SE** | **p (unadj)** | **OR lower** | **OR upper** | **p (BH-adj)** |
| Headache | 0.61 | 0.18 | 0.01 | 0.43 | 0.87 | 0.19 |
| Fear | 0.70 | 0.20 | 0.08 | 0.46 | 1.05 | 0.74 |
| Loss of initiative | 1.48 | 0.26 | 0.14 | 0.88 | 2.53 | 0.74 |
| Incontinence | 0.49 | 0.48 | 0.14 | 0.18 | 1.24 | 0.74 |
| Stiffness | 0.54 | 0.48 | 0.21 | 0.19 | 1.39 | 0.74 |
| Slow | 1.34 | 0.24 | 0.21 | 0.84 | 2.18 | 0.74 |
| Depression | 0.74 | 0.26 | 0.26 | 0.44 | 1.24 | 0.74 |
| Get lost | 0.86 | 0.15 | 0.32 | 0.63 | 1.15 | 0.74 |
| Diplopia | 0.65 | 0.42 | 0.32 | 0.27 | 1.51 | 0.74 |
| Dizziness | 0.76 | 0.27 | 0.33 | 0.44 | 1.31 | 0.74 |
| Hypesthesia and tingling in skin | 1.43 | 0.38 | 0.35 | 0.67 | 3.16 | 0.74 |
| Muscle pull | 1.60 | 0.51 | 0.35 | 0.60 | 4.68 | 0.74 |
| Decreasing weight | 0.77 | 0.31 | 0.42 | 0.41 | 1.44 | 0.78 |
| Hemiplegia | 1.41 | 0.45 | 0.44 | 0.59 | 3.61 | 0.78 |
| Cognitive impairment | 1.12 | 0.20 | 0.56 | 0.75 | 1.67 | 0.88 |
| Orientation | 1.15 | 0.24 | 0.56 | 0.71 | 1.86 | 0.88 |
| Loss of strength | 1.20 | 0.37 | 0.61 | 0.58 | 2.54 | 0.89 |
| Word finding disorder | 0.93 | 0.17 | 0.68 | 0.66 | 1.31 | 0.89 |
| Memory problems | 0.94 | 0.14 | 0.72 | 0.71 | 1.26 | 0.89 |
| Gloom | 0.95 | 0.15 | 0.76 | 0.70 | 1.29 | 0.89 |
| Delusions | 0.93 | 0.28 | 0.80 | 0.53 | 1.63 | 0.89 |
| Nausea | 1.07 | 0.27 | 0.80 | 0.62 | 1.84 | 0.89 |
| Anhedonia | 0.86 | 0.63 | 0.81 | 0.23 | 3.12 | 0.89 |
| Nightmares | 1.01 | 0.56 | 0.98 | 0.33 | 3.16 | 0.99 |
| Hallucination | 1.00 | 0.18 | 0.99 | 0.69 | 1.44 | 0.99 |
